# Prevalence and Risk Factors of Psychiatric Symptoms Among Older People in England During the COVID-19 Pandemic: a Latent Class Analysis

**DOI:** 10.1007/s11469-022-00820-2

**Published:** 2022-04-26

**Authors:** Emma Curran, Michael Rosato, Finola Ferry, Gerard Leavey

**Affiliations:** Bamford Centre for Mental Health and Wellbeing, Ulster University, Coleraine, Northern Ireland UK

## Abstract

The COVID-19 pandemic has affected mental health and social connections. Older people may be disproportionately affected, placing them at increased risk for complex mental ill-health outcomes and quality of life undermined by anxiety and depression. Understanding gender differences in the determinants of anxiety and depression symptoms is crucial to policy and practice. This study aims to examine gender-specific symptom subtypes (and subthreshold symptoms) in an older English population sampled during the COVID period, in relation to their socio-demographic, social, and health circumstances. The sample comprises all individuals aged 50 years or older and included in the *English Longitudinal Study of Ageing* COVID-19 sub-study conducted during June–July 2020. Latent class analysis (LCA) defined indicative sample subgroups of clinically relevant anxiety and depression. Multinomial logistic regression assessed associations between socio-demographic characteristics, health and social care indicators, loneliness, and pre-pandemic mental ill-health. LCA derived three classes of self-reported depression and anxiety: for females (1) comorbid depression and anxiety (19.9% of the sample), (2) depression and subthreshold anxiety (31.6%), and (3) no or low symptoms of depression and anxiety (48.5%), and for males (1) comorbid depression and anxiety (12.8%), (2) subthreshold anxiety and depression (29.6%), and (3) no or low depression and anxiety (57.6%). Multinomial logistic regression analyses indicate that compared to those with low/no mental health symptoms, severity of pandemic-era mental ill-health was positively associated with pre-pandemic mental health levels, worry over finances, having access to essentials, loneliness, and access to health and social care services. Findings support the persistence of comorbidity of both depression and anxiety in the pandemic period. Results may inform government health strategy on interventions to prevent social isolation and mitigate the effects of the pandemic on deteriorating mental health in older people who may be more susceptible.

The impact of social isolation on the mental health of older people has been exacerbated by the ongoing COVID-19 pandemic. In England, closure of non-essential shops, offices and public spaces, restrictions on non-essential travel, and regulated self-isolation and quarantine measures were announced by the UK Government on 23 March 2020. Threats from serious infection, enforced isolation, and reduced access to health services and support groups may undermine mental health (Kola et al., n.d.). Previous findings from the *English Longitudinal Study of Ageing* (ELSA) indicate that older people are at high risk (Zaninotto et al., n.d.). While these findings are informative, they are mostly descriptive, unable to disentangle the subtleties and interactions that impact psychiatric vulnerability among older people. Social disconnection puts older people at greater risk of depression and anxiety, and this risk to health and social well-being is uneven (Santini et al., n.d.). Pre-pandemic, health, and social disparities were reported among older people who had limited social contacts, more emotional distress, a higher risk of loneliness, and limited access to healthcare (Curran et al., n.d.; Stickley & Koyanagi n.d.). During the pandemic and lockdown, these challenges are likely to have intensified and COVID-19 studies note increased prevalence of depression, anxiety, and loneliness, with vulnerability highlighted in women, minority ethnic populations, people of lower socioeconomic status, and people with pre-existing physical and mental illness (Salari et al., n.d.). Extended pandemic quarantine periods may impact on these (Ismael et al., n.d.) which, as noted, may differ as a function of sex and age (Vindegaard & Benros n.d.). During the pandemic, health inequalities are expected to become more pronounced, placing socio-economically disadvantaged and marginalised groups at increased risk (Coronini-Cronberg et al., n.d.). Longer-term consequences associated with social isolation, loneliness, and depression include cognitive decline (Bu et al., n.d.) and incident dementia (Suárez-González et al., n.d.).

## Study Aims

ELSA is an ongoing prospective population–based cohort study of adults aged 50 years and over living in England (Zaninotto & Steptoe n.d.). Using an extension of the main study carried out after onset of the pandemic, our aim is to evaluate the emotional and social experience of older people during the early months of the pandemic, and to understand how it has impacted their well-being. We hypothesise that for varying sub-classes of anxiety-depression, reduced social functioning and reduced access to healthcare can contribute to more severe mental ill-health outcomes in the pandemic phase. We address these questions by carrying out Latent Class Analysis on clinically representative mental health indicators of depression and anxiety and associations with health service access, loneliness, and pre-existing (pre-COVID) mental health, all associated with the precarity of ageing and premature mortality (Craig n.d.).

## Method

### Sample

The data came from the first wave of the ELSA COVID-19 sub-study (an extension of the regular ELSA sample) (Steptoe et al., n.d.), carried out in June–July 2020 to investigate the socioeconomic and psychological impact of the COVID-19 pandemic. The response rate was high (75%). The sample included all 7040 members of the COVID-19 sub-study. Analyses were weighted to match the latest population estimates for age, sex, and region in England and account for non-response to the ELSA COVID-19 sub-study survey. All respondents provided informed consent. Data used in this study can be obtained upon free registration at the UK Data service (https://beta.ukdataservice.ac.uk/datacatalogue/series/series?id=200011). Further information regarding the sample design and data collection methods can be found on the study website (https://www.elsa-project.ac.uk/).

### Measures

#### Outcomes

We focused on two mental health outcomes, symptoms of depression and anxiety. Depression symptoms were ascertained using the 8-item *Centre for Epidemiological Studies Depression* (CESD-8) scale, which measures eight different symptoms of depression (e.g. *felt depressed*, *everything.. was an effort*, *sleep was restless*). This scale was validated against gold-standard psychiatric interviews with good sensitivity and specificity (Radloff n.d.). Anxiety was measured using the 7-item *generalised anxiety disorder* scale (GAD-7), which evaluates the presence of various symptoms of generalised anxiety disorder (GAD) (e.g. *feeling nervous, anxious or on edge*, *not being able to stop or control worrying*). This scale is a reliable screening tool for assessing GAD and its severity in both research and clinical practice (Spitzer et al., n.d.). A dichotomous (no/yes) response was created for each item of the CESD-8 and GAD-7.

#### Sociodemographic Characteristics

The main sociodemographic characteristics considered in the analysis were: age, sex, ethnicity, housing tenure, and partner in the household. Age comprised three groups: 50–64, 65–74, and 75 + years. Sex and ethnicity were binary fields: men/women, and minority ethnic group/other, respectively. Housing tenure included four categories (owns outright; owns with mortgage; rents; lives rent free).

#### Covariates

##### Care Needs

Respondents were asked *Since the coronavirus outbreak started have your care needs been met…*, with five response categories (*all the time*; *most of the time*; *some of the time*; *hardly ever*; and *no care needs)*.

##### Access to Health and Social Care Services

Respondents were asked *Since the outbreak have you been able to access the community health and social care services.. you need, for instance a dentist, podiatrist, nurse, counselling or personal care?* with four response categories (*yes; no*; *no attempt to make contact*; *and no need to make contact*).

##### Worry About Finances and Essentials

Respondents were asked *How worried.. are you about*: (a) *your future financial situation*; and (b) *not having essential items during the coronavirus outbreak* — each with three response categories (*not worried*; *somewhat worried*; *very worried*).

##### Loneliness

Was assessed using the 3-item revised *University of California (UCLA) Loneliness scale* (Hughes et al., n.d.) (*How often do you feel*: *lack of companionship*; *left out*; *isolated from others*), and an additional item asking participants *how often they feel lonely*. Each question was rated on a 3-point scale (*hardly ever/never*; *some of the time*; *often*). These individual scores were summed (range 1–12), with higher values indicating greater loneliness.

##### Depression at ELSA Wave 9 (Pre-pandemic)

Depressive symptoms were ascertained at wave 9 (2018/2019) using the CESD-8 scale. A dichotomous (no/yes) response was asked of each item, with responses accumulated resulting in a total CESD-8 score between zero (no symptoms) and eight (all eight symptoms). We then generated a binary variable using a standard cut-off of four or more symptoms to identify likely cases of clinical depression, equivalent to the conventional threshold of sixteen or higher on the full 20-item CESD scale (Karim et al., n.d.).

### Statistical Analyses

Table 1 reports separately for males and females the levels of reported depressive or anxiety symptoms as measured in the depression (CES-D) and anxiety (HADS-A) from the ELSA COVID-19 sub-study.

**Table 1:** Frequency of depressive or anxiety symptoms and percentages within gender as measured in the depression (CES-D) and anxiety (HADS-A), reported in the COVID-19 Sub-study of the English Longitudinal Study of Ageing (ELSA)

	Total sample 7040 *N* (%)	Males 3060 (43.5%) *N* (%)	Females 3980 (57.5%) *N* (%)
Questions from the CES-D^$^ depression subscale			
You felt depressed	1232 (17.5)	432 (14.1)	800 (20.1)
You felt that everything you did was an effort	1684 (23.9)	585 (19.1)	1099 (27.6)
Your sleep was restless	3159 (44.9)	1203 (39.3)	1956 (49.3)
You were not happy	1113 (15.8)	387 (12.7)	726 (18.4)
You felt lonely	1209 (17.2)	342 (11.2)	867 (21.9)
You did not enjoy life	1291 (18.3)	431 (14.1)	860 (21.8)
You felt sad	411 (5.8)	121 (17.5)	290 (30.5)
You could not get going	1931 (27.4)	666 (21.8)	1265 (31.9)
Questions from the HADS-A^%^ anxiety subscale			
Feeling nervous, anxious or on edge	2572 (36.5)	832 (27.3)	1740 (43.9)
Not being able to stop or control worrying	1855 (26.3)	564 (18.5)	1291 (32.6)
Worrying too much about different things	2546 (36.2)	838 (27.5)	1708 (43.2)
Trouble relaxing	2414 (34.3)	857 (28.1)	1557 (39.5)
Being so restless that it is hard to sit still	1721 (24.4)	603 (19.8)	1118 (28.3)
Becoming easily annoyed or irritable	2688 (38.2)	1043 (34.2)	1645 (41.5)
Feeling afraid as if something awful might happen	1868 (26.5)	589 (19.3)	1279 (32.3)

^$^Depressive symptoms assessed using the 8-item *Centre for Epidemiologic Studies-Depression* (CES-D) which assesses symptoms experienced in the 7 days preceding the survey (Radloff, 1977)

^%^Anxiety symptoms assessed using the anxiety subscale of the *Hospital Anxiety and Depression Scale* (HADS-A), which measures the presence of anxiety symptoms with no specific time frame (Zigmond & Snaith, 1983)

#### Latent class analysis (LCA)

This can identify discrete groups based on endorsement of items reported. Using data from the ELSA COVID-19 sub-study LCA detected, separately for males and females, clusters of symptoms reported on the CES-D and HADS-A. Fit indices, based on the depression and anxiety questions, were evaluated for LCA models in Table 2. Solutions for six classes were estimated, with log-likelihoods, information criteria (IC) and classification accuracy examined. To identify the best solution, we considered a combination of statistical criteria, model parsimony, interpretability, meaningfulness, and the need for theory and judgement. In Table 2, the AIC, BIC, and SSABIC were lower in the 3-class solution, compared to two classes, VLMR and Entropy further indicated the 3-class solution as optimal; therefore, it was selected for analysis. For females, results of LCA are reported in Table 2: here, results indicated a 3-class solution as preferable, suggested by a lower AIC, BIC, and SSABIC in the 3-class solution (compared with two classes), both the Entropy and VLMR test indicate the 3-class solution as optimal.

**Table 2:** Fit indices of the LCA to profile depression and anxiety symptoms in males and females

Model 2a	Log	Free	AIC	BIC	SSABIC	LRT	*ρ*	Entropy
1 Class males	− 23,414	30	46,889	47,067	46,971			
2 Class males	− 18,464	61	37,050	37,412	37,218	9859	0.00	0.93
*3 Class males	**− 17,619**	**92**	**35,422**	**35,967**	**35,675**	**1683**	**0.00**	**0.89**
4 Class males	− 17,296	123	34,838	35,567	35,177	643	0.75	0.88
5 Class males	− 17,071	154	34,451	35,365	34,876	446	0.26	0.86
6 Class males	− 16,931	185	34,233	35,331	34,743	278	0.76	0.84
Model 2b	Log	Free	AIC	BIC	SSABIC	LRT	*ρ*	Entropy
1 Class females	− 34,488	15	69,006	69,100	69,052			
2 Class females	− 26,933	31	53,928	54,122	54,024	14,996	0.00	0.92
*3 Class females	**− 25,836**	**47**	**51,767**	**52,062**	**51,913**	**2176**	**0.00**	**0.87**
4 Class females	− 25,400	63	50,926	51,321	51,121	866	0.10	0.81
5 Class females	− 25,196	79	50,550	51,045	50,794	405	0.09	0.79
6 Class females	− 25,024	95	50,238	50,834	50,532	340	0.02	0.79

*Log* log likelihood function, maximum likelihood estimation; *Free* free parameters, *LRX2* likelihood ratio chi-square, *AIC* Akaike information criteria, *BIC* Bayesian information criteria, *SSABIC* sample size adjusted BIC, *LRT* Lo-Mendell-Rubin adjusted likelihood ratio test. ^*^Denotes most parsimonious model

#### LCA 3 Step Approach

Following identification of satisfactory LCA models describing patterns of anxiety and depression, we explored the interrelationships between them. One of the difficulties in testing structural relationships between latent classes lies in controlling for any uncertainty associated with the assignment of the classes (Nylund-Gibson et al., n.d.). Treating classes as if they were qualities measured without error and uncertainty may bias model parameters and therefore subsequent results. We applied a recently developed solution to this problem which involves a three step approach: firstly, estimating satisfactory latent class models for the patterns of interest (patterns of anxiety and depression symptoms); secondly, assigning individuals to the most likely class; and finally, assigning individuals into a latent class considered as a nominal indicator of their latent class, with measurement parameters fixed at values which take account of the measurement error in class assignment (Curran et al., n.d.; Nylund-Gibson et al., n.d.). This approach has the advantage of estimating the measurement models in a separate step, whereby the estimation is neither influenced by heterogeneity between the indicators of the different processes of interest, nor by other covariates. We tested associations by regressing the latent classes of depression and anxiety onto the covariates of interest. Because latent classes are nominal variables, we ran multinomial logistic regressions, indicating odds ratios and confidence intervals associated with sub-groups of depression and anxiety.

## Results

This analysis is based on an older population (aged 50 years or more) entering late adulthood at the beginning of the pandemic — 7040 respondents: 3060 (43.5%) males and 3980 (57.5%) females. Table 1 reports the gender-specific prevalence of symptoms of either depression or anxiety: here, females generally report higher proportions on all components of the scales.

**Fig. 1:**
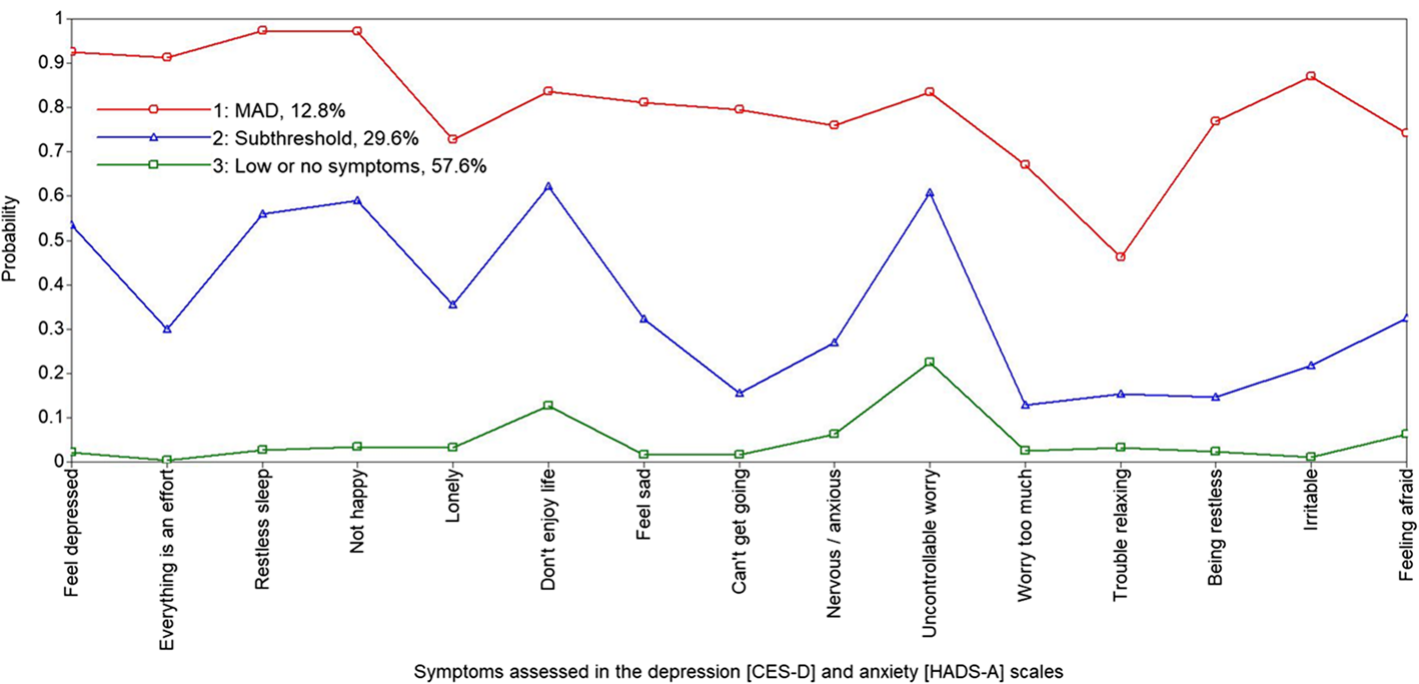
The 3-class solution (males) — this comprises (1) comorbid depression and anxiety (MAD) (12.8% of the sample), with a high probability of reporting symptoms of both depression and anxiety; (2) subthreshold anxiety and depression (29.6%); and (3) no or low depression and anxiety symptoms (57.6%)

**Fig. 2:**
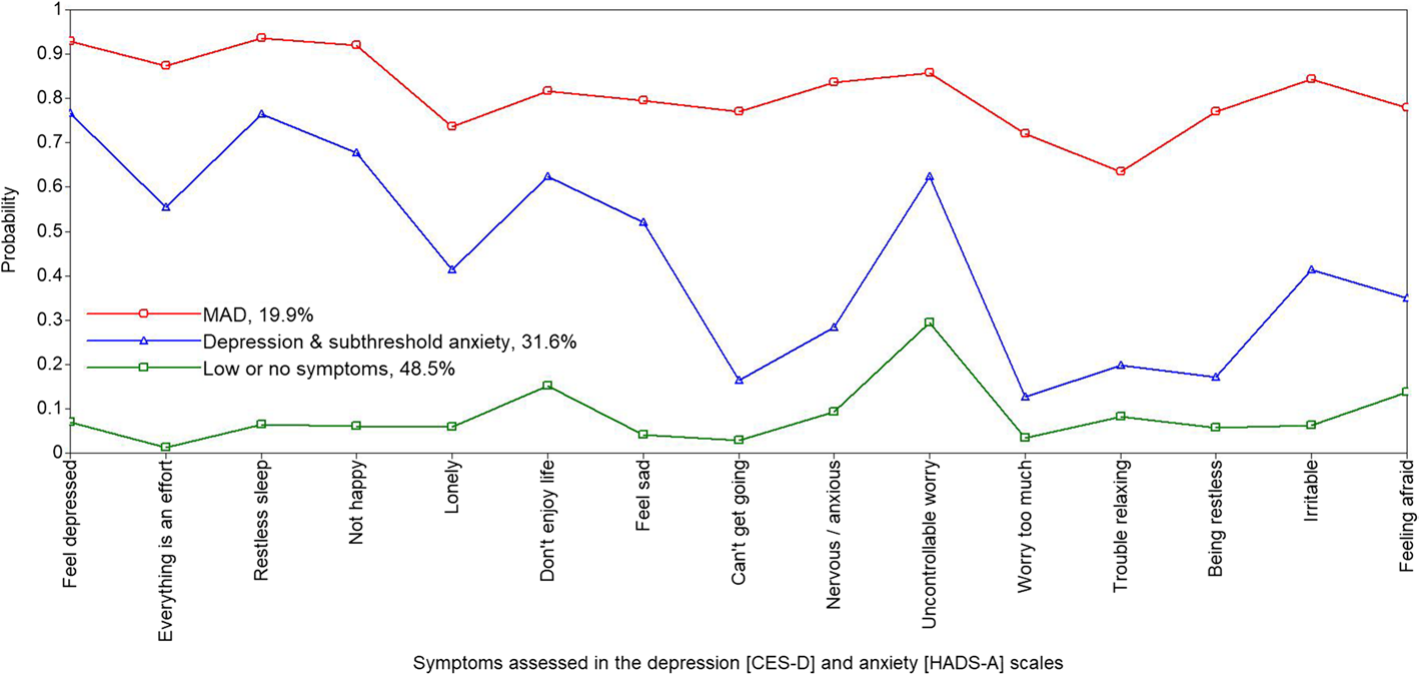
The 3-class solution (females) — this comprises (1) comorbid depression and anxiety (MAD) (19.9% of the sample), with a high probability of reporting symptoms of depression and anxiety; (2) depression and subthreshold anxiety (31.6%); and (3) no or low depression and anxiety symptoms [48.5%]

Figures 1 and 2 depict sample proportions and plot-estimated probabilities for each solution. The models, similar in distribution, distinguish clear patterns for symptom expression, based on (1) *comorbid (or mixed) anxiety and depression* (MAD) — 13% and 20% of males and females respectively, (2) *depression and subthreshold* (or borderline) *anxiety* — 30% of males and 32% of females, and (3) *no or low depression or anxiety* (49% of females and 58% of males). This latter group were the reference group for comparisons in the subsequent regression modelling.

### Socio-demographic Factors, Personal Worries, Access to Care, Loneliness, and Pre-pandemic Mental Health

Tables 3 and 4 (males and females respectively) each present two sets of incrementally adjusted models (one each for *comorbid depression/anxiety* and *subthreshold depression and anxiety*), with each compared against *no/low levels of depression/anxiety*. Each set comprises two models — M1 (adjusted for concerns about service use and personal worries over personal resources) and M2 (further adjusted for personal isolation and pre-pandemic depression). Additionally, the models are adjusted for ethnicity. While both age and housing tenure are included as adjustment factors in the models, for brevity are not presented (they are available on request).

**Table 3:** Male LCA groups and regression adjusted for age and housing tenure

		Comorbid depression and anxiety (MAD)		Subthreshold depression and anxiety	
		M1	M2	M3	M4
Ethnicity	Not minority ethnic Minority ethnic group	1.00 0.49 (0.13, 1.91)	1.00 0.39 (0.12, 1.29)	1.00 0.56 (0.28, 1.11)	1.00 *0.42 (0.21, 0.87)
Health care needs met	No care needs Always met Mostly met Sometimes met Hardly ever met	1.00 *2.65 (1.59, 4.42)*6.28 (2.91, 13.55)*6.28 (2.18, 18.08) *11.81 (4.34, 32.13)	1.00 *2.22 (1.23, 3.99) *4.89 (1.42, 16.82) 1.19 (0.27, 5.18) *11.35 (3.27, 39.47)	1.00 *1.43 (1.01, 2.01) 0.90 (0.47, 1.75) 0.47 (0.14, 1.56) 1.61 (0.42, 6.15)	1.00 1.33 (0.93, 1.91) 0.90 (0.43, 1.89) 0.27 (0.06, 1.12) 3.07 (0.73, 12.96)
Accessed community HSC services	Didn’t need to contact Didn’t attempt contact Yes No	1.00 1.03 (0.62, 1.72) 0.71 (0.41, 1.24) *1.66 (1.08, 2.55)	1.00 0.91 (0.55, 1.50) 0.88 (0.51, 1.54) *1.62 (1.06, 2.47)	1.00 *1.86 (1.29, 2.68) 1.40 (0.94, 2.09) 1.14 (0.80, 1.62)	1.00 *1.75 (1.17, 2.59) 1.51 (0.98, 2.32) 1.02 (0.70, 1.49)
Worry about finances	Not at all worried Somewhat worried Very worried	1.00 1.25 (0.78, 1.99) *6.45 (3.86, 10.79)	1.00 1.16 (0.70, 1.93) *6.05 (3.36, 10.91)	1.00 1.22 (0.92, 1.63) *2.83 (1.93, 4.16)	1.00 1.11 (0.82, 1.49) *2.72 (1.81, 4.07)
Worry about having essentials	Not at all worried Somewhat worried Very worried	1.00 *1.94 (1.24, 3.03) *6.86 (3.77, 12.49)	1.00 1.50 (0.89, 2.52) *3.34 (1.37, 8.13)	1.00 *2.06 (1.54, 2.75) *2.90 (1.66, 5.05)	1.00 *1.92 (1.42, 2.59) *2.23 (1.08, 4.60)
Partner in household	No Yes		1.00 *3.08 (1.52, 6.24)		1.00 *1.68 (1.10, 2.56)
UCLA loneliness	Scale 0–12		*2.12 (1.81, 2.49)		*1.64 (1.44, 1.86)
Depression at wave 9 (pre C-19)	No Yes		1.00 *9.62 (4.43, 20.91)		1.00 *2.43 (1.17, 5.05)

^*^Denotes significant *p*-value

**Table 4:** Female LCA groups and regression adjusted for age and housing tenure

		Comorbid depression and anxiety (MAD)		Depression and subthreshold-anxiety	
		M1	M2	M3	M4
Ethnicity	Not minority ethnic Minority ethnic group	1.00 *0.49 (0.25, 0.98)	1.00 *0.37 (0.16, 0.83)	1.00 1.09 (0.68, 1.75)	1.00 1.08 (0.62, 1.88)
Health care needs met	No care needs Always met Mostly met Sometimes met Hardly ever met	1.00 *1.64 (1.11, 2.44) *2.18 (1.37, 3.47) *5.77 (2.51, 13.25) *5.38 (1.88, 15.42)	1.00 1.36 (0.84, 2.18) *1.84 (1.07, 3.15) 2.40 (0.80, 7.19) 1.75 (0.32, 9.70)	1.00 *1.59 (1.17, 2.15) 1.41 (0.85, 2.33) 0.93 (0.30, 2.88) 1.53 (0.44, 5.27)	1.00 *1.58 (1.14, 2.20) 1.33 (0.81, 2.20) 0.53 (0.11, 2.59) 1.42 (0.33, 6.08)
Accessed community HSC services	Didn’t need to contact Didn’t attempt contact Yes No	1.00 *2.11 (1.52, 2.93) *2.11 (1.44, 3.09) *1.64 (1.13, 2.38)	1.00 *1.62 (1.10, 2.38) *1.65 (1.02, 2.67) 1.20 (0.78, 1.85)	1.00 *1.38 (1.05, 1.82) 0.96 (0.68, 1.38) 1.19 (0.89, 1.59)	1.00 1.21 (0.89, 1.64) 0.83 (0.57, 1.20) 1.00 (0.73, 1.36)
Worry about finances	Not at all worried Somewhat worried Very worried	1.00 *1.45 (1.05, 2.00) *4.25 (3.01, 5.99)	1.00 *1.52 (1.04, 2.23) *2.74 (1.80, 4.17)	1.00 1.12 (0.89, 1.41) *2.51 (1.89, 3.34)	1.00 1.21 (0.95, 1.54) *2.44 (1.79, 3.33)
Worry about having essentials	Not at all worried Somewhat worried Very worried	1.00 *2.71 (1.84, 3.97) *6.64 (3.33, 13.26)	1.00 *2.19 (1.55, 3.10) *3.60 (2.00, 6.50)	1.00 *1.63 (1.30, 2.06) *2.07 (1.31, 3.27)	1.00 *1.52 (1.18, 1.95) *1.78 (1.07, 2.94)
Partner in household	No Yes		1.00 *2.05 (1.42, 2.96)		1.00 *2.03 (1.57, 2.62)
UCLA loneliness	Scale 0–12		*2.13 (1.95, 2.33)		*1.36 (1.27, 1.47)
Depression at wave 9 (pre C-19)	No Yes		1.00 *3.96 (2.62, 5.99)		1.00 *2.04 (1.40, 2.97)

^*^Denotes significant *p*-value

### Comorbid Depression and Anxiety (MAD)

Persons reporting *comorbid depression and anxiety* (compared with their non-compromised peers) were more likely to be younger and in rented accommodation; females (Tables 4) were less likely to be from a minority ethnic background (OR = 0.37: 95%CI = 0.16, 0.83), and both males and females (Tables 3 and 4) were more likely to report problems of unmet health care needs — in M1 (highlighting personal concerns), while females recorded five-fold excesses in relation to pandemic-related unmet need this disappeared after including indicators of social isolation/prior depression (M2); for males however, the recorded excesses of M1 attenuated, remaining for those who felt their needs were *hardly ever met* (OR = 11.35: 3.27, 39.49). Both males and females with *comorbid depression/anxiety* reported being worried about finances (OR = 6.05: 3.36, 10.91 and OR = 2.74: 1.80, 4.17 respectively in the fully adjusted models) and having access to essentials (OR = 3.34: 1.37, 8.68 and OR = 3.60: 2.00, 8.13 respectively) during the pandemic. Finally, in the fully adjusted models those with *comorbid depression/anxiety* were more likely to record higher levels of loneliness, despite having a partner in the household (OR = 2.12: 1.81, 2.49; OR = 2.13: 1.95, 2.33 for males and females respectively), as well as being associated with pre-pandemic depression (OR = 9.62: 4.43, 20.91; OR = 3.96: 2.62, 5.99 respectively).

### Depression and Subthreshold Anxiety

*Depression and subthreshold anxiety* was less likely among both older individuals and females living in *rented accommodation*. While health care needs were not problematic for either males or females with *depression/subthreshold anxiety*, high levels of worry about finances (OR = 2.72: 9%CI = 1.81, 4.07 and 2.44: 1.79, 3.33 for males and females respectively) and having access to essentials (OR = 2.23: 1.08, 4.60 and OR = 2.44: 1.79, 3.33 respectively) were recorded. In the fully adjusted models (M2), higher levels of loneliness, despite being more likely to have a partner in the household, were more likely among males and females (OR = 1.64: 1.44, 1.86 and OR = 1.36: 1.27, 1.47, respectively), as well as being associated with pre-pandemic depression (OR = 2.43: 1.17, 5.05; OR = 2.04: 1.40, 2.97 respectively).

## Discussion

Half of this older population reported poor mental health outcomes during the early months of the pandemic. Our findings highlight that depression and anxiety often occur as comorbid disorders (Curran et al., n.d.). Mental health symptoms were not uniform across gender or the different age groups, as shown in the LCA models which indicate levels of severity. Those in the younger age groups (aged 50–64 years) were more likely to report greater severity of mental ill-health, than those aged 65 and over. This finding, discussed in a previous study, is linked to possible challenges within a transitional life-stage for some around retirement (Curran et al., n.d.; Hawkley & Kocherginsky n.d.). However, for others, it highlights uncertainty and financial fears due to the pandemic and associated employment instability. It has been noted that pandemic related economic downturn will significantly impact short and long term health (Vahia et al., n.d.). As physical and mental health are interlinked, our findings are in line with the negative effects of economic instability, lack of access to essential services, and the deleterious consequences for mental health (Tilburg et al., n.d.). During the 2008–2010 recession, each ten percent increase in unemployment for males was significantly associated with a significant increase suicides (1.4%: 0.5 to 2.3%) (Barr et al., n.d.). Our findings show that persistent mental health issues are compounded during the pandemic period by financial worries, related to accessing housing essentials like heating and food.

Vulnerable groups with health conditions are most likely to endure the cascade of negative effects of the crisis. Our study stresses that those disproportionately impacted also have limited access to health and social care services, access to care at home and have fears about financial security, all associated with the greatest severity in poor mental health. This finding reflects distress following lockdown measures and quarantine (stay at home orders, social distancing, uncertainty about finances, access to services and essential commodities, and interruption of care) (Zaninotto et al., n.d.). Currently, older people are at higher risk of more isolation and worse outcomes after infection, thus are more likely to need access to health and social care services (Zaninotto et al., n.d.).

Ethnic minority status was protective for females reporting comorbid depression and anxiety, an unexpected finding, considering current evidence that those identifying as minority ethnic groups record both disproportionately higher risks of mental ill-health and being adversely affected by COVID-19 in the UK and USA (Khunti et al., n.d.). Ethnic disparities include reduced access to UK health systems, which disproportionately effects migrants, critical care admissions, unequal access to services, and premature mortality (Modesti et al., n.d.). However, the single aggregate measure (of ethnicity) used in ELSA may mask true differences that exist between ethnic groups. Additionally, because of undocumented migrants having limited access to healthcare and services, and not likely to be picked up in large epidemiological studies (Mathur et al., n.d.), they may be more likely to be disproportionately affected by the current pandemic and numbers of COVID cases.

Our results highlight the interplay between mental health and social well-being, with levels of loneliness higher among people with depression and anxiety and a clear positive gradient evident between symptom severity and loneliness. People reporting mental health issues during the pandemic report being lonely, despite having a partner living at home. It is known that age is a risk factor for limiting social connections, isolation, and loneliness. Previous research on loneliness, suggests that the negative impacts of social isolation are more profound for males than females (Cacioppo et al., n.d.) and that marital quality plays a crucial role (Warner & Kelley-Moore n.d.).

## Conclusion

Our research provides a picture of the mental health effects of the pandemic during the early months in 2020. In our study, pre-pandemic data indicates previous depression at wave 9 is an indicator of poor mental health during the current pandemic. Examining longer-term determinants of mental ill-health and the consequences that these have on the elderly are important. Depressive and anxiety symptoms are highly associated with the incidence of a range of chronic conditions and premature mortality; associations reported in our study show that there are sub-groups of high risk older people whose health may deteriorate more generally, compared to other older people in the population.

Furthermore, during the early months of the pandemic, symptom severity appears exacerbated due to the disruption to health and social care services, access to care at home, concerns over financial security, and feelings of loneliness. Provision of access to mental health services should be a priority, either delivered online, through mobile phone technologies or importantly over the phone for older people with limited digital resources. Our findings highlight a need to bolster the mental health of older people in England through facilitating access to health and social support services, until in person contact is feasible. Thus far, government priority has focused on limiting the spread of disease, largely through enforcing shielding and social distancing for high-risk groups considered vulnerable; however, a longer-term strategy should consider the cost of isolation on health and social wellbeing for older people, as it is likely to increase strain and exacerbate poor outcomes in those already at risk (Steinman et al., n.d.).

### Limitations

A limitation of our study relates to the time period for when data collection took place, which occurred when the first lockdown was easing. Our results may be underreporting mental health problems that could have been higher during the period of April through to May 2020 (Fancourt et al., n.d.). Regardless, our study demonstrates the importance directing focus towards offsetting the adverse mental health implications of the pandemic on older people living in the community.
